# Gene Signatures Detect Damaged Liver Sinusoidal Endothelial Cells in Chronic Liver Diseases

**DOI:** 10.3389/fmed.2021.750044

**Published:** 2021-10-20

**Authors:** Stefaan Verhulst, Elise Anne van Os, Vincent De Smet, Nathalie Eysackers, Inge Mannaerts, Leo A. van Grunsven

**Affiliations:** Liver Cell Biology Research Group, Vrije Universiteit Brussel, Brussel, Belgium

**Keywords:** LSEC, acute liver injury, single cell RNA sequencing (scRNAseq), primary cells, NAFLD (non-alcoholic fatty liver disease), non-alcoholic steatohepatitis (NASH)

## Abstract

Liver sinusoidal endothelial cells have a gatekeeper function in liver homeostasis by permitting substrates from the bloodstream into the space of Disse and regulating hepatic stellate cell activation status. Maintenance of LSEC's highly specialized phenotype is crucial for liver homeostasis. During liver fibrosis and cirrhosis, LSEC phenotype and functions are lost by processes known as capillarization and LSEC dysfunction. LSEC capillarization can be demonstrated by the loss of fenestrae (cytoplasmic pores) and the manifestation of a basement membrane. Currently, no protein or genetic markers can clearly distinguish healthy from damaged LSECs in acute or chronic liver disease. Single cell (sc)RNA sequencing efforts have identified several LSEC populations in mouse models for liver disease and in human cirrhotic livers. Still, there are no clearly defined genesets that can identify LSECs or dysfunctional LSEC populations in transcriptome data. Here, we developed genesets that are enriched in healthy and damaged LSECs which correlated very strongly with healthy and early stage- vs. advanced human liver diseases. A damaged LSEC signature comprised of *Fabp4/5* and *Vwf/a1* was established which could efficiently identify damaged endothelial cells in single cell RNAseq data sets. In LSECs from an acute CCl_4_ liver injury mouse model, *Fabp4/5* and *Vwf/a1* expression is induced within 1–3 days while in cirrhotic human livers these 4 genes are highly enriched in damaged LSECs. In conclusion, our newly developed gene signature of damaged LSECs can be applicable to a wide range of liver disease etiologies, implicating a common transcriptional alteration mechanism in LSEC damage.

## Introduction

Liver sinusoidal endothelial cells (LSECs) comprise about 15–20% of the total number of liver cells and line the sinusoidal lumen of the liver sinusoids. LSECs are highly specialized endothelial cells characterized by fenestrae and lack of a basement membrane (1) making these cells the most permeable cells in the mammalian body (2). LSEC permeability is important for liver function as it permits plasma, solutes, and small substrates such as albumin (3) and insulin (4) to diffuse from the blood toward the parenchymal cells. Besides working as a filter and first barrier of the liver, these cells have other functions such as the production of coagulation factor VIII (5), antigen presentation (6, 7), leukocyte recruitment and endocytosis of virus particles (8), oxidized LDL (9) and immunocomplexes by the abundant expression of multiple scavenger receptors (10). Expression of specific scavenger receptors and other characteristic proteins can vary across the liver acinus (11, 12).

Maintenance of the specialized LSEC phenotype is essential for liver homeostasis (13). During liver injury LSECs can become dysfunctional, characterized by the loss of fenestrae and the appearance of a basement membrane, also known as capillarization (4, 14–16). LSECs can contribute to liver regeneration and healing by orchestrating an angiocrine response that can lead to a pro-regenerative response after an acute injury or to a maladaptive pro-fibrotic response after chronic injury, which in turn leads to fibrosis (17). Moreover, LSECs are described to have a gatekeeper function in liver fibrosis as differentiated LSECs promote HSC quiescence, and restoration of LSEC differentiation can prevent fibrosis progression and accelerate fibrosis regression (18). Although it is known that LSECs play an important role in the response to acute and chronic liver injury, research on the transcriptomic and phenotypic change of LSECs during acute and chronic injury is still limited. In addition, identification of LSECs using genetic/protein markers is still quite controversial (19) as there is no unique marker that characterizes LSECs (11, 13) apart from fenestrae and the absence of a basement membrane. The identification of damaged LSECs in an acute or chronic setting is even more challenging. Recently, specific markers for LSECs in healthy livers have been described, such as CD32b (20), CLEC4G (21), LYVE1 (22), STAB2 (23) in addition to the more controversial endothelial cell (EC) markers VWF and CD31 which are upregulated in LSECs during disease (13, 19). However, currently electron microscopy is still the golden standard for identification of damaged LSECs (loss of fenestrae). The recent use of single-cell transcriptomics (scRNAseq), performed on both healthy and diseased human and mouse livers, has identified several heterogeneous hepatic cell populations, including LSECs (12, 21, 24–26). These publicly available data sets present bioinformatic opportunities to define LSEC populations more efficiently in both healthy and diseased livers, independent of the etiology or background.

In this study, we developed healthy- and damaged LSEC enriched gene sets and signatures using healthy and cirrhotic human liver scRNAseq data and newly generated datasets from healthy and acutely injured mouse livers. These LSEC genesets and signatures can identify the health status of LSECs in mouse and human bulk transcriptome or scRNAseq data from chronic or acute liver diseases. Using these gene sets, we demonstrate that LSECs are dysfunctional in multiple end-stage liver diseases and that LSECs are quickly damaged upon an acute liver injury. These results highlight the important role of LSECs in liver pathophysiology.

## Materials and Methods

### Animals

All methods and protocols were carried out according to the approved guidelines of the Vrije Universiteit Brussel (VUB, Belgium) and according to European Guidelines for the Care and Use of Laboratory Animals. Animal experiment protocols were approved by the Ethical Committee of Animal Experimentation of the Vrije Universiteit Brussel (VUB, Belgium, 14-212-4). BalbC mice aged 11–14 weeks were housed in a controlled environment in conventional cages and were allowed food and water *ad libitum*. Acute liver injury in BalbC mice was induced by a single intraperitoneal injection with 15 μl carbon tetrachloride (CCl_4_, 87031, Sigma-Aldrich, St. Louis, MO, USA) and 85 μl mineral oil (Sigma-Aldrich, St. Louis, MO, USA) per 30 g bodyweight. Blood, total liver and cells were collected from healthy mice and after 1, 3 and 7 days of CCl_4_ administration. Mice were anesthetized using 100 μL Dolethal^®^ (Vetoquinol, France). Analysis of alanine aminotransferase (ALT) was performed using a SPOTCHEM EZ SP-4430 (A.Menarini Diagnostics, The Netherlands). At the start and end of the experiment mice were weighted. Daily observation of the mice showed only a mild effect on animal welfare.

### LSEC Isolation From Mice

Non-parenchymal cells (NPCs) were retrieved as previously described (27). Red blood cell lysis (Miltenyi Biotec, Germany) was performed, and NPCs were washed with PBS + 0.1% Bovine Serum Albumin (BSA). NPCs were resuspended in BPE buffer (PBS with 5% BSA and 2 mM EDTA) with 1 ul anti-mouse Fc block™ (Becton-Dickinson, Belgium) reagent added per 10^7^ cells for 10 min at 4°C. Cells were washed and incubated in 600 μL PBS+0.1% BSA with 5 μl CD32-PE (ab30357, Abcam, UK), 2 μL CD45-FITC (11-0451-85, eBioscience, USA) and 10 μl F4/80 Alexa-647 per 10^7^ cells (MF48021, Life Technologies) for 15 min at 4°C. After incubation with the antibodies, cells were washed and resuspended in a buffer solution without calcium and supplemented with DNase I (3:1, 10104159001, Roche, Switzerland) before cell isolation using FACS (FACS Aria IIu, BD Biosciences, Belgium). FACS was used to sort viable cells (negative selection based on propidium iodide) and LSECs were selected and sorted based on a positive signal for CD32 (27–29) and a negative signal for UV, F4/80, CD45. CD32b is expressed in all LSECs across the liver sinusoid (Supplementary Figure 1A) (30). Potential doublets with HSCs, KCs, and immune cells were excluded (cfr. Supplementary Figure 1B. Utmost right FACS plot with circled LSEC population). Stainings were performed on cytospins after isolation and showed a high purity (95%) of LSECs using this sorting strategy (Supplementary Figure 1C).

### RNA Preparation and Sequencing

Total RNA was extracted from FACS-isolated LSECs using ReliaPrep RNA Cell Miniprep System (Z6012, Promega, USA), RNA concentrations and quality measurements were performed using a Bioanalyzer 6000. Preparation of samples and sequencing, using Clontech SMARTseq v4 kit (R400752, Takara, Japan) and NovaSeq S2 (2 × 100 bp), was performed by the BRIGHTcore of the Vrije Universiteit Brussel. Single-end sequencing was run on Illumina NextSeq 500 High.

### Immunofluorescence

Mouse liver tissues were fixed with formalin for 48 h at 4°C. Liver tissues were stored in 70% EtOH and were used for sectioning (Leica, The Netherlands) of 100 μm liver sections in 4% UltraPure™ Low Melting Point Agarose (Invitrogen, USA) using a vibratome (Leica, The Netherlands). Sections were kept in 70% EtOH until usage. Upon usage sections were rehydrated in 50% EtOH for 10 min and rinsed for 10 min with PBS. For permeabilization, sections were incubated with PBS + 0.2% Triton for 20 min at room temperature. After permeabilization, sections were washed two times with PBS and blocked with 3% BSA-PBS for 2 h at room temperature. Sections were incubated overnight at room temperature with the following primary antibodies; Lyve1 (2 μg/mL, AF2125, R&D systems, Canada), Ki67 (0.5 μg/mL, 14-5698-82, Thermofisher, USA) and CD32b (10 mg/mL, AF2125, R&D systems, Canada). PHEM buffer (10 mM PIPES, 25 mM HEPES, 10 mM EGTA, 2 mM MgCl2^*^6H2O) was used for CD32b staining instead of PBS in all steps. Vibratome sections were washed three times with PBS for 10 min and were incubated for 1 h with the following secondary antibodies (1:200); Donkey-anti-goat Alexa488 (A11055, Thermofisher, USA) and Donkey-anti-rat Alexa 647 (ab150155, Abcam, UK). Sections were washed three times with PBS, incubated for 10 min with 70% EtOH and then incubated with 1% Sudan Black (199664, Sigma-Aldrich, Belgium) in 70% EtOH. Sections were rinsed with PBS and mounted with Mowiol (9002-89-5, Sigma-Aldrich, Belgium) with DAPI (D9564, 10 μg/mL, Sigma-Aldrich, Belgium) and visualized by EVOS M7000 (Thermofischer, USA) and Zeiss Axioscan (Zeiss, Germany). Quantification was performed with HALO 3.1 image analysis platform (Indica labs Inc., USA).

### Immunohistochemistry

Liver tissues were embedded in paraffin, sliced in 5 μm sections and deparaffinized with Xylene. For H&E stainings sections were rehydrated, washed with PBS and counterstained with Harris Hematoxylin (1:10 Roth, Newport Beach, CA, USA) before being rinsed with acid water followed by 10 min wash with tap water. Sections were incubated with eosin for 5 min, shortly rinsed, dehydrated and mounted with DPX mounting medium (Sigma-Aldrich, Belgium). For Collagen 4 staining, sections were rehydrated, washed with PBS-0.05%Tween (PBST) and endogenous peroxidase was quenched with 3% H_2_O_2_ in methanol. Samples were washed three times with PBST for 5 min and incubated with 2% BSA-PBS for 1 h at room temperature. Col4 antibody (2 μg/ml, ab6586, Abcam, UK) was dissolved in 1% BSA-PBS and incubated overnight at 4°C. Sections were washed and incubated with Dako EnVision+ System- HRP Labeled Poly (K4003, Dako, Denmark) for 30 min at room temperature. Sections were washed with PBST, incubated with DAB substrate for 3 min at room temperature. Finally, samples were rinsed, counterstained with Harris Hematoxylin (1:10) and mounted with DPX mounting medium (Sigma-Aldrich, Belgium) and imaged visualized with Leica Aperio CS2 (Leica, The Netherlands). Quantification was performed with Orbit image analysis (31).

### Bioinformatics

#### scRNAseq Analysis

Raw counts of scRNAseq data from healthy and diseased livers of Ramachandran et al. (GSE136103) (25), MacParland et al. (GSE115469) (24), Aizarani (GSE124395) (21), Xiong et al. (GSE129516) (26), and Terkelsen et al. (GSE145086) (32) was downloaded from GEO-NCBI database and imported into RStudio (https://www.rstudio.com). General scRNAseq analysis for quality controls, normalization, clustering and multidimensional reduction was performed using the default pipeline of R package Seurat (33). Identification of different cell clusters was performed using markers from the original publications and visualized in a UMAP plot.

#### Differential Expressed Genes in scRNAseq Data

Genes differentially expressed between two populations were identified using the *findmarker* function within R package Seurat with fold changes larger than 2.

#### Downstream Analysis for scRNAseq

Creation and visualization of different gene signatures (LSEC signatures) by upset plots was performed by the usage of R packaged UpSetR. Gene ontology analysis based on biological processes was analyzed using R package clusterProfiler for all gene signatures. The *AddmoduleScore* function in Seurat (version 4) was used to quantify gene signature scores of all LSEC signatures for each cell population. The gene signature score represents the average expression of all genes of the healthy or damaged LSEC gene signature within a cell population subtracted by the average expression of randomly selected genes within the same population.

#### Whole Transcriptome Analysis

Paired-end sequencing on RNA of LSECs isolated using FACS from healthy and CCl_4_ treated mice generated a fastq file for each sample. A quality control was performed before and after trimming using FastQC (www.bioinformatics.babraham.ac.uk/projects/fastqc) and AfterQC (34) followed by mapping all reads using STAR (35) to the mouse genome GRCm38.p6. Assembly was performed on every hit using StringTie and further analyzed by R package DESeq2 (36) for normalization and statistical analysis. Principle component analysis was performed using basic R functions and visualized by R package ggplot2. The expression of a selection of genes was validated using qPCR (Supplementary Figure 2A). qPCR was performed as previously described (27) and primers used for qPCR are displayed in Supplementary Figure 2B. For microarray data, CEL files were imported using R packages oligo (37) or affy (38) and normalized by Robust Multichip Average (RMA) algorithm.

#### Gene Set Enrichment Analysis (GSEA)

GSEA Subramanian et al. (39) was performed on normalized counts using molecular signature databases Reactome, Biocarta and KEGG pathways. GSEA for bulk seq of LSECs was performed by comparing all groups (LSECs isolated from mouse livers after 1, 3, and 7 days CCl_4_ injection) to LSECs from healthy mouse livers. All enriched pathways with a NES (normalized enrichment score) higher than 1 or lower than −1 with FDR lower than 0.25 were imported in Cytoscape and transformed into a network using EnrichmentMap (40). Pathways clustered together were named manually, based on overlapping functions, following the protocol of Reimand et al. (41). Pathway clusters that change over time were manually summarized into a hypothetical graph created in Illustrator, based on the number of pathways within a cluster and changes after CCl_4_ injection. GSEA using LSEC enriched gene sets was performed on normalized counts of healthy and liver diseases or on LSECs isolated from healthy or CCl_4_ recovered livers. Following comparisons were performed to analyse LSEC signatures in advanced diseased livers vs. control groups: Hepatitis B (HBV) F3-4 vs. HBV F0-1 (GSE84044) (42), non-alcoholic steatohepatitis (NASH) F3–F4 vs. F0–F1 (GSE49541) (43), alcoholic steatohepatitis (ASH) vs. alcoholic steatosis liver (GSE103580) (44), advanced cirrhosis vs. healthy (GSE6764) (45), advanced hepatocellular carcinoma (HCC) vs. normal tissue (GSE6764) (45).

#### Data Availability

Bulk RNAseq data of isolated LSECs after CCl_4_ treatment has been deposited in the GEO public data base under accession number: GSE180366.

### Statistics

One-tailed Kruskal Wallis with Dunnett's multiple comparisons test was applied for the statics of ALT measurements, CD32b, Lyve1 and Lyve1/Ki67 stainings. Calculations were made using GraphPad Prism 9. Ns > 0.05, ^*^P ≤ 0.05, ^**^P ≤ 0.01.

## Results

### Enriched Genes in LSECs From Healthy and Cirrhotic Livers Identify LSECs in Advanced Cirrhotic Liver Diseases

**Figure 1:**
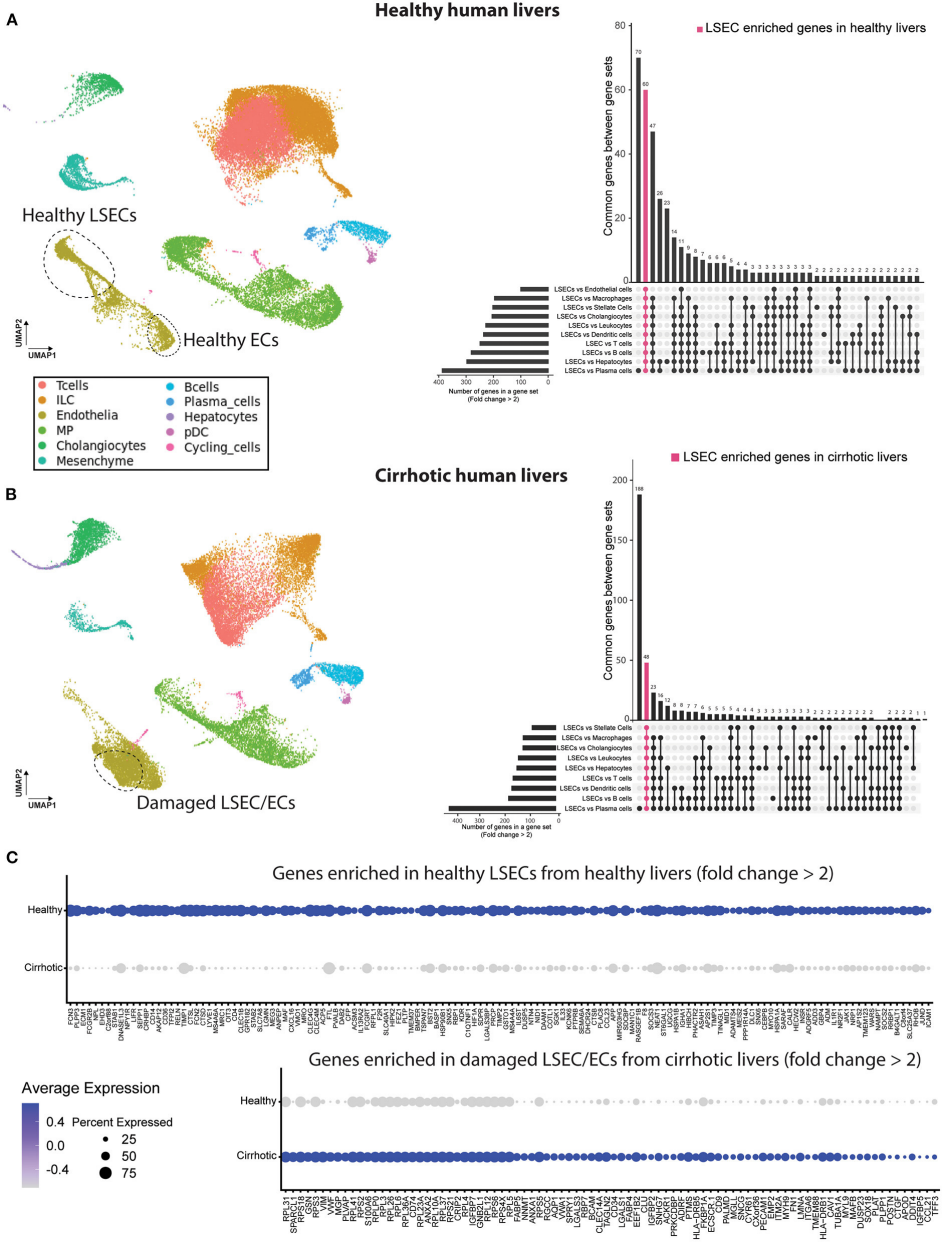
Identification of genes higher expressed in LSECs in healthy and cirrhotic human livers. Left a UMAP plot of scRNAseq data of healthy **(A)** and cirrhotic **(B)** liver cells (25). Right an upset plot of differentially expressed genes (fold change > 2) in LSECs or damaged LSEC/ECs compared to all other cell types. Pink color represents LSEC enriched genes in healthy livers (60 genes) or damaged LSEC/EC enriched genes in cirrhotic livers (48 genes). **(C)** Dotplot of differentially expressed genes between LSECs of healthy and cirrhotic livers with fold change >2. All results were obtained with the use of the dataset of Ramachandran et al. (25).

**Figure 2:**
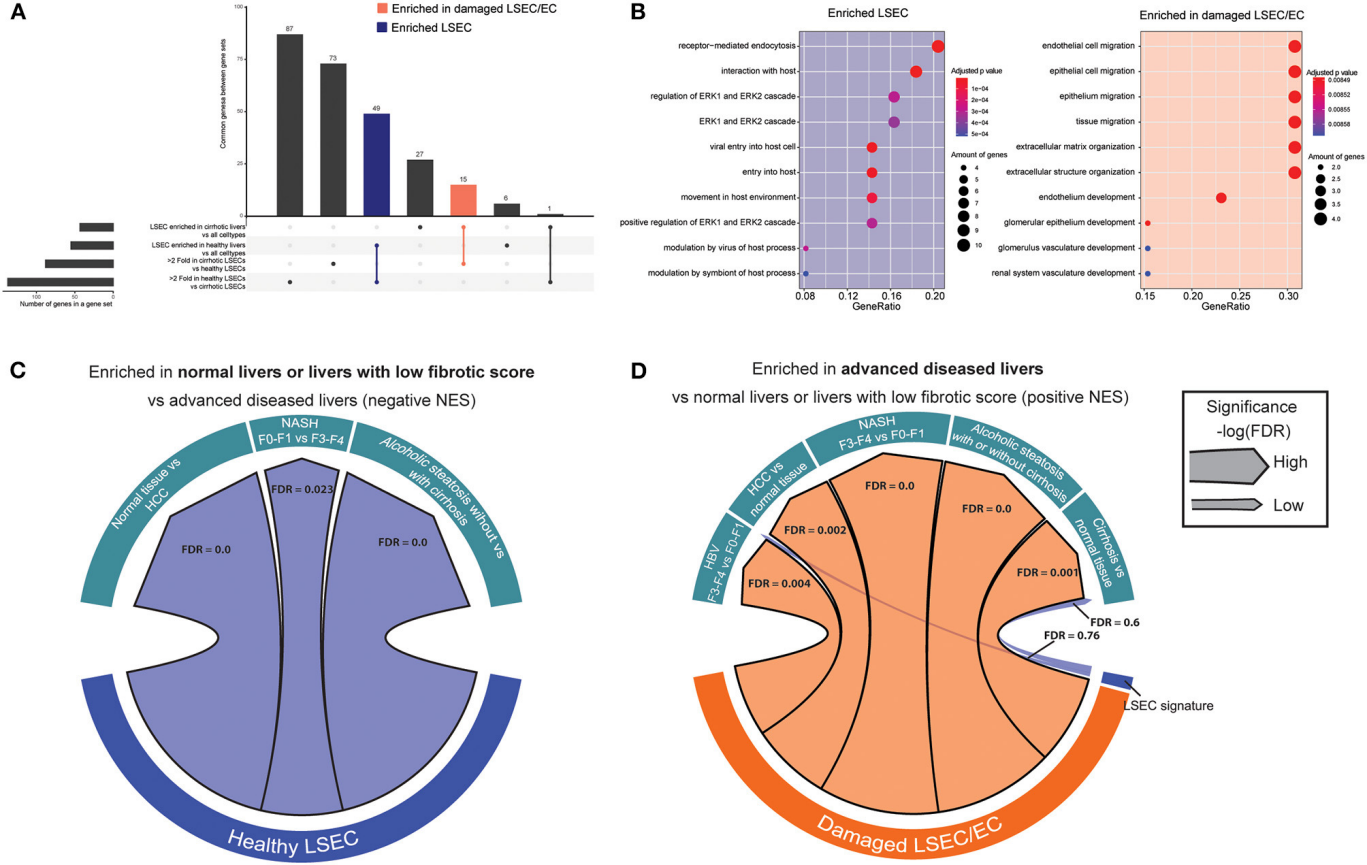
Enriched gene sets in LSECs from healthy and cirrhotic livers identify healthy and damaged LSECs in chronic liver diseases. **(A)** Upset plot that combines genes that are differentially expressed between LSECs from healthy livers and damaged LSEC/ECs from cirrhotic livers and genes enriched in LSECs when compared to all other cell types in healthy and cirrhotic livers. Results were obtained with the use of the dataset of Ramachandran et al. (25). **(B)** Gene ontology analysis (biological processes) on enriched genes from LSECs and damaged LSECs/ECs. **(C,D)** Chord diagram of GSEA analysis [significance, –log(FDR)] of enriched genes from LSEC or LSEC/ECs in advanced liver diseases: cirrhotic livers (vs healthy livers) Wurmbach et al. (45), ASH (vs alcoholic steatosis) Trépo et al. (44), HCC (vs. normal tissue) Wurmbach et al. (45), NASH (F3–4 vs. F0–1) Murphy et al. (43) and HBV (F3–4 vs. F0–1) Wang et al. (42).

To identify the presence of healthy or dysfunctional LSECs in RNA profiling data sets from human or mouse livers we set out to identify genes that are enriched in LSECs from healthy or cirrhotic livers. To this end, we used scRNAseq data of healthy and cirrhotic human livers reported by Ramachandran et al. (25). First, we identified LSECs and ECs expressing known LSEC and EC markers in healthy livers (Figure 1A; Supplementary Figure 3). Next, we identified an additional cell population which was not present in the endothelial cell population of healthy livers (Figure 1B). These cells were CD34^+^PLVAP^+^VWA1^+^ positive which strongly resembled the scar-associated endothelial cell population identified by Ramachandran et al. (25). These cells were restricted to cirrhotic livers, expressed pro-fibrogenic genes and displayed an immunomodulatory phenotype (25). We refer to this population as damaged LSEC/ECs (Figure 1B) as some of the markers expressed in this population show a sinusoidal expression pattern in cirrhotic livers (25) but damaged ECs cannot be excluded. Subsequently, we defined genes that were higher expressed in healthy LSECs or damaged LSEC/EC population compared to all other liver cells (endothelial cells, macrophages, stellate cells, cholangiocytes, innate lymphoid cells (ILC), dendritic cells, T and B cells, hepatocytes and plasma cells) from healthy and cirrhotic livers with a fold change of at least two, and every gene should be expressed in at least 50% of cells within the healthy LSEC or damaged LSEC/EC population. This resulted in, respectively, 60 and 48 genes that were higher expressed in LSECs from healthy- or cirrhotic human livers in comparison to other liver cell types (Figures 1A,B). To identify genes that can further distinguish LSECs from healthy or diseased livers, we performed differential expression analysis between both populations resulting in a list of genes expressed higher in healthy LSECs compared to damaged LSEC/ECs from cirrhotic livers (Figure 1C). By combining genes that are enriched in LSECs from healthy or cirrhotic livers vs. other cells with genes that are higher expressed in one of the conditions vs. the other, we could create two genesets: a geneset for LSECs from healthy livers (*n* = 48) and a geneset for damaged LSEC/EC from cirrhotic livers (*n* = 15) (Figure 2A; Supplementary Table 1). Next, we performed gene ontology analyses to summarize the overlap in biological functions of genes included in each geneset. Genes that were enriched in healthy LSECs were part of GOs that are related to scavenging function and viral entry, both important characteristics of LSECs (46, 47). Genes that are enriched in damaged LSEC/EC belong to GOs that are related to dysfunctional LSECs, such as vascular development, migration and matrix organization, which are typical features of liver fibrosis (48, 49) (Figure 2B).

Next, we wondered whether we could use these gene sets to visualize an enrichment of damaged LSECs/ECs in microarray gene expression data from human livers. We therefore performed gene set enrichment analysis (39, 50) (GSEA) with the two LSEC gene sets on microarray gene expression data from human livers with different etiologies to identify the presence of healthy or damaged LSECs/ECs in advanced cirrhotic liver diseases. Figure 2C shows that gene sets that were highly expressed in healthy LSECs were substantially enriched in transcriptomes of healthy livers and diseases livers with early stage liver fibrosis (F0–F1). Gene sets that were highly expressed in damaged LSEC/ECs were enriched in cirrhotic livers (vs healthy livers) (45); ASH (vs alcoholic steatosis) (44), HCC (vs normal tissue) (45), NASH (F4–F3 vs. F0–F1) (43) and HBV (F3–4 vs. F0–1) (42) (Figure 2D). Taken together, our analysis suggests that LSECs transform into a more damaged endothelial cell phenotype in all advanced liver diseases that we investigated.

### Dynamic Response of LSECs to CCl_4_-Induced Acute Liver Injury

**Figure 3:**
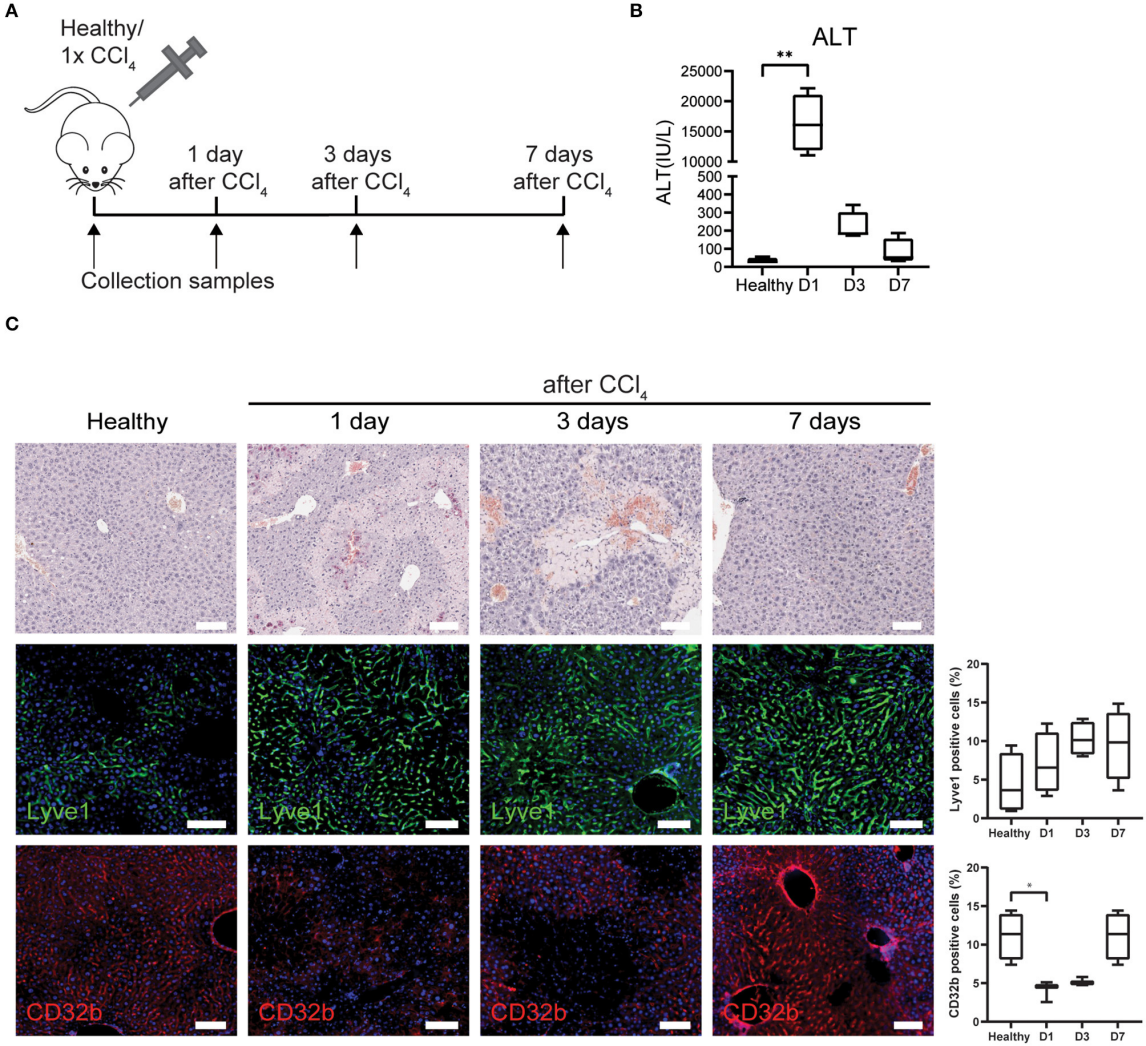
LSECs in healthy livers or acute liver injury. **(A)** Isolation of LSECs from healthy livers and from livers after 1, 3, and 7 days after CCl_4_ injection. **(B)** ALT levels of healthy mice and mice that received CCl_4_ **P ≤ 0.01. **(C)** Immunohistochemistry with hematoxylin eosin staining and immunofluorescence staining and quantification of Lyve1 and CD32b (*P ≤ 0.05) on livers from healthy mice or mice that received CCl_4_ (bar = 100 μm).

LSECs play a crucial role in the regenerative response after an acute injury that can either lead to liver regenerative or a maladaptive fibrotic response (17). Yet, all studies and datasets we have used so far only reflected chronic liver injury. Therefore, we wanted to know whether the gene sets could also demonstrate LSEC phenotype changes after an acute injury. To this end, acute liver injury in mice was induced with a single dose of CCl_4_ and livers were collected at 1, 3, and 7 days after injection (Figure 3A). Blood analysis shows acute liver injury (high ALT levels) at 24 h after a single dose of CCl_4_, which decreases to baseline levels at day 7 (Figure 3B). Hematoxylin eosin staining shows necrotic areas that appear at 1 day and are more pronounced after 3 days demonstrating that liver injury is still present at that time point (Figure 3C). However, after 1 week the liver appears to have recovered from the injury. When we further examine LSECs through staining, we see an increased trend of Lyve1 protein levels indicating that LSECs are still present and sinusoids are intact. However, we observed a temporary loss of CD32b expression after 1 and 3 days of CCl_4_ treatment, indicating at least partial LSEC dysfunction which is restored after 1 week.

**Figure 4:**
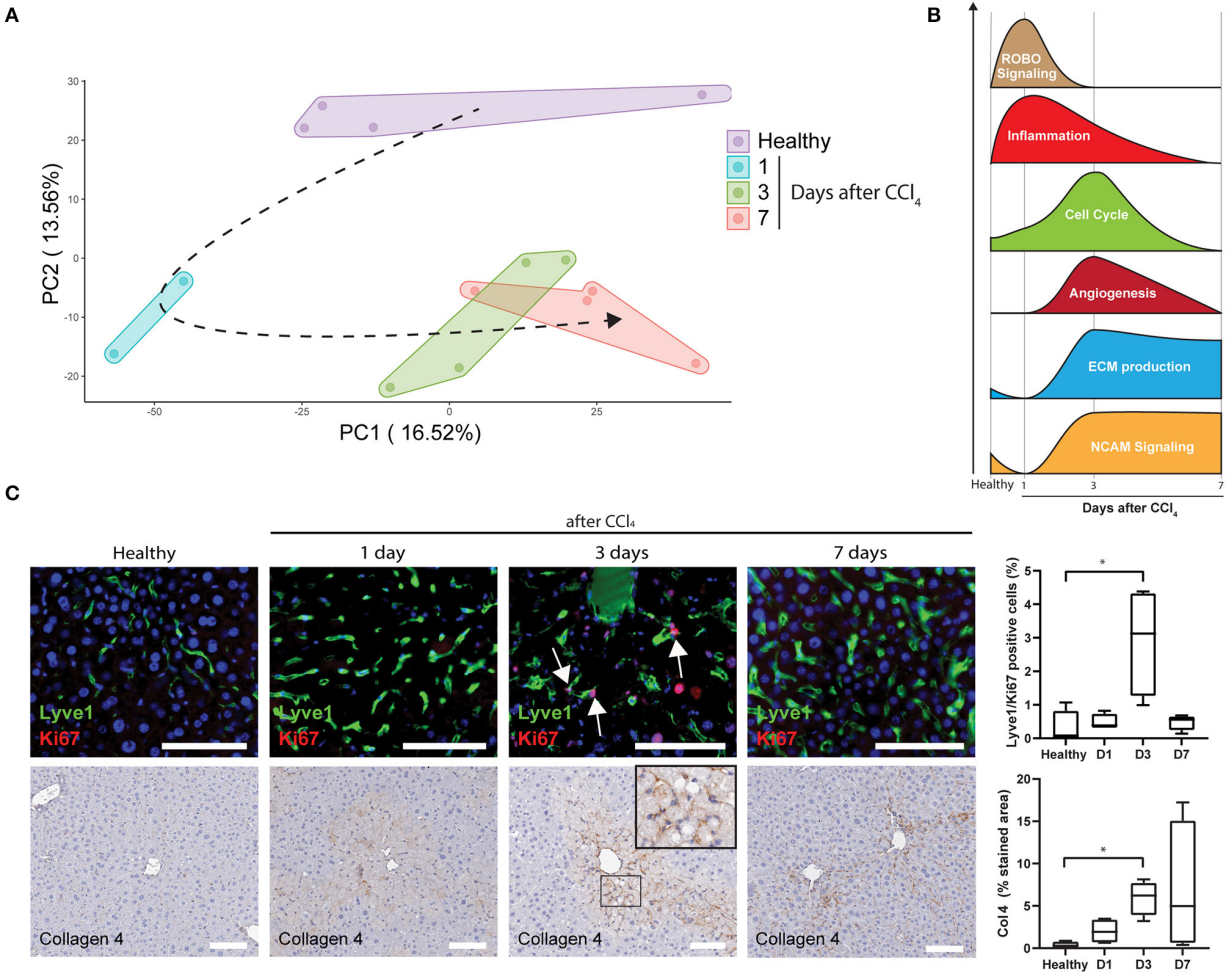
Upregulated pathways in LSECs during acute liver injury **(A)** PCA of LSECs from healthy livers and livers after an acute injury by CCl_4_ administration. **(B)** Schematic representation of pathway analysis from LSECs isolated after CCl_4_ administration. **(C)** Immunofluorescence staining of Lyve1/Ki67, immunohistochemistry staining and quantification of Collagen 4 (*P ≤ 0.05) and Lyve1/Ki67 positive cells (*P ≤ 0.05) on healthy livers and livers after CCl_4_ administration (bar = 100 μm).

To further analyse LSECs after acute liver injury, livers were collected and LSECs were isolated via FACS at 1, 3, and 7 days after CCl_4_ administration (Figure 3A; Supplementary Figure 1B) and transcriptome analysis was performed on the freshly isolated LSECs. Four samples were included for each condition apart from LSECs after 1 day of CCl_4_-treatment, because 2 samples did not meet the quality standards for RNA sequencing (low RIN values). Although this reduces the statistical power, still more than 2,000 genes were differentially expressed when compared to healthy LSECs (Supplementary Figure 4). Principal component analysis (PCA) demonstrates separated clusters for each timepoint indicating a change in LSEC transcriptome after exposure to CCl_4_ (Figure 4A). Interestingly, LSECs appear not to restore to the healthy LSEC cluster after CCl_4_ induced injury, indicating that LSECs after 1 week CCl_4_ have a different phenotype compared to healthy LSECs. Next, pathway analysis was performed on LSECs from CCl_4_-treated livers compared to healthy LSECs (40). All enriched pathways were clustered in Cytoscape (Supplementary Figure 5) and graphically represented in Figure 4B. Shortly after the induction of acute liver injury, several pathways related to ROBO signaling and inflammation become significantly enriched (NES > 1, FDR <0.25). After 3 days, pathways involved in angiogenesis, ECM (extra cellular matrix) production and cell cycle are induced. Interestingly, a considerable amount of cell cycle pathways are strongly active after 3 days of CCl_4_ but seem to become inactive again after 7 days. This was confirmed by the presence of Ki67^+^Lyve1^+^ positive LSECs in livers 3 days after CCl_4_, indicating that indeed LSECs are proliferating at day 3, but not anymore after 7 days (Figure 4C). Pathways regarding ECM production were elevated after 3 days of CCl_4_ and remained elevated after 7 days. NCAM signaling, important for the inhibition of fibroblast growth factor signaling (51), shows a similar trend. One of the dysregulated ECM genes is Collagen 4 which has been described to be produced by LSECs (52–54). Upon acute injury we indeed see an induction of Collagen 4 expression on day 3, which shows a sinusoidal pattern (Figure 4C).

### Generation and Validation of an LSEC- and a Damaged LSEC Signature

**Figure 5:**
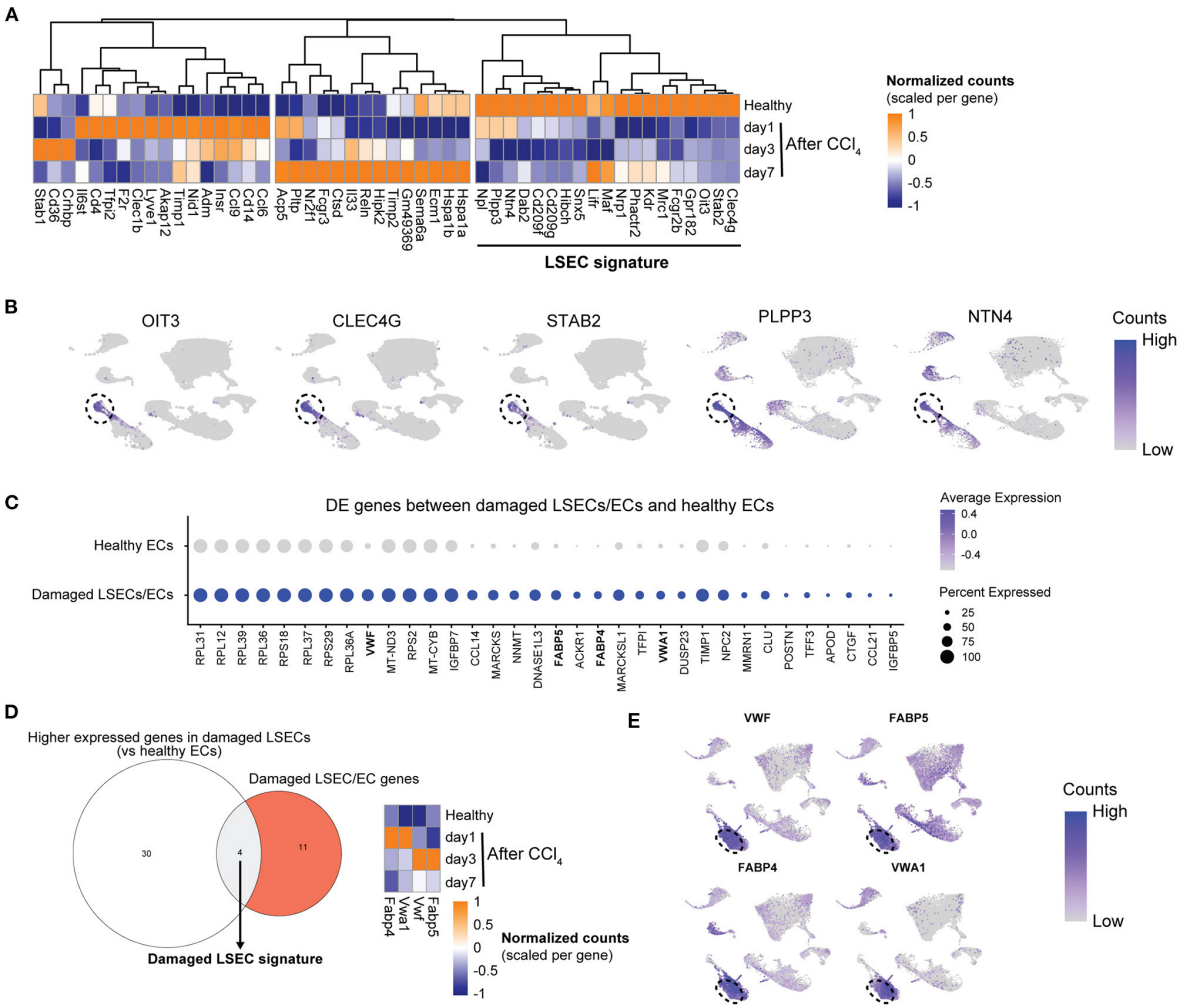
Development of an LSEC and a damaged LSEC gene signature. **(A)** Heatmap of the enriched healthy human LSEC genes in LSECs from healthy and acutely injured mouse livers. The genes used for the LSEC signature are underlined. **(B)** UMAP plot of Ramachandran et al. (25) dataset with gene expressions in healthy livers for LSEC signature genes with the LSEC population marked by the dotted line. **(C)** Dot plot of differentially expressed genes between damaged LSEC/EC population and healthy ECs from Ramachandran et al. (25). **(D)** Venn diagram of genes higher expressed in damaged LSECs from **(C)** and damaged LSEC/EC signature. The damaged LSEC signature is represented by the overlapping region. Heatmap of LSEC signature genes in LSEC from healthy and acutely injured livers. **(E)** UMAP plot of Ramachandran et al. (25) dataset with gene expression from damaged LSEC signature genes in cirrhotic livers. Damaged LSEC population is marked by the dotted line.

To compare human with mouse LSEC dysfunction, we analyzed the expression of human LSEC gene sets (Figures 1, 2) in mouse LSECs after an acute CCl_4_-induced liver injury. Genes that are enriched in healthy human LSECs show diverse expression patterns in mouse LSECs after acute liver injury (Figure 5A). Typical LSEC genes such as *STAB2* and *CLEC4G* are downregulated upon liver injury, in contrast to genes such as *LYVE1, CLEC1B*, and *CD36* which are upregulated at early timepoints, indicating that these genes cannot always discriminate healthy LSECs from damaged LSECs. Genes that were expressed higher in healthy LSECs were selected for the generation of a restricted healthy LSEC signature that should identify healthy LSECs in mice and human samples. This signature contains both novel (*PLPP3, NTN4* and *OIT3*) and well-established (*CLEC4G* and *STAB2*) genes for LSECs which show a high expression in healthy human LSECs (Figure 5B). To generate also a more restricted gene signature that can specifically identify LSECs in damaged livers instead of both damaged LSECs and ECs, we first identified genes that were differentially expressed in the damaged LSEC population in comparison to healthy endothelial cells (Figure 5C). These differentially expressed genes were compared to previously identified enriched gene sets from damaged LSECs/ECs (Figure 2A) which resulted in a damaged LSEC signature that contained only four genes: *Fabp4/5* and *Vwf/a1* (Figure 5D). These four genes were all upregulated in CCl_4_-induced liver injury after 1 day or 3 days. Moreover, these four genes are highly expressed in the damaged human LSEC population of the Ramachandran et al. (25) data set (Figure 5E). The expression of two healthy and damaged LSEC signature genes were validated using qPCR and confirmed the RNAseq data (Supplementary Figure 2A).

**Figure 6:**
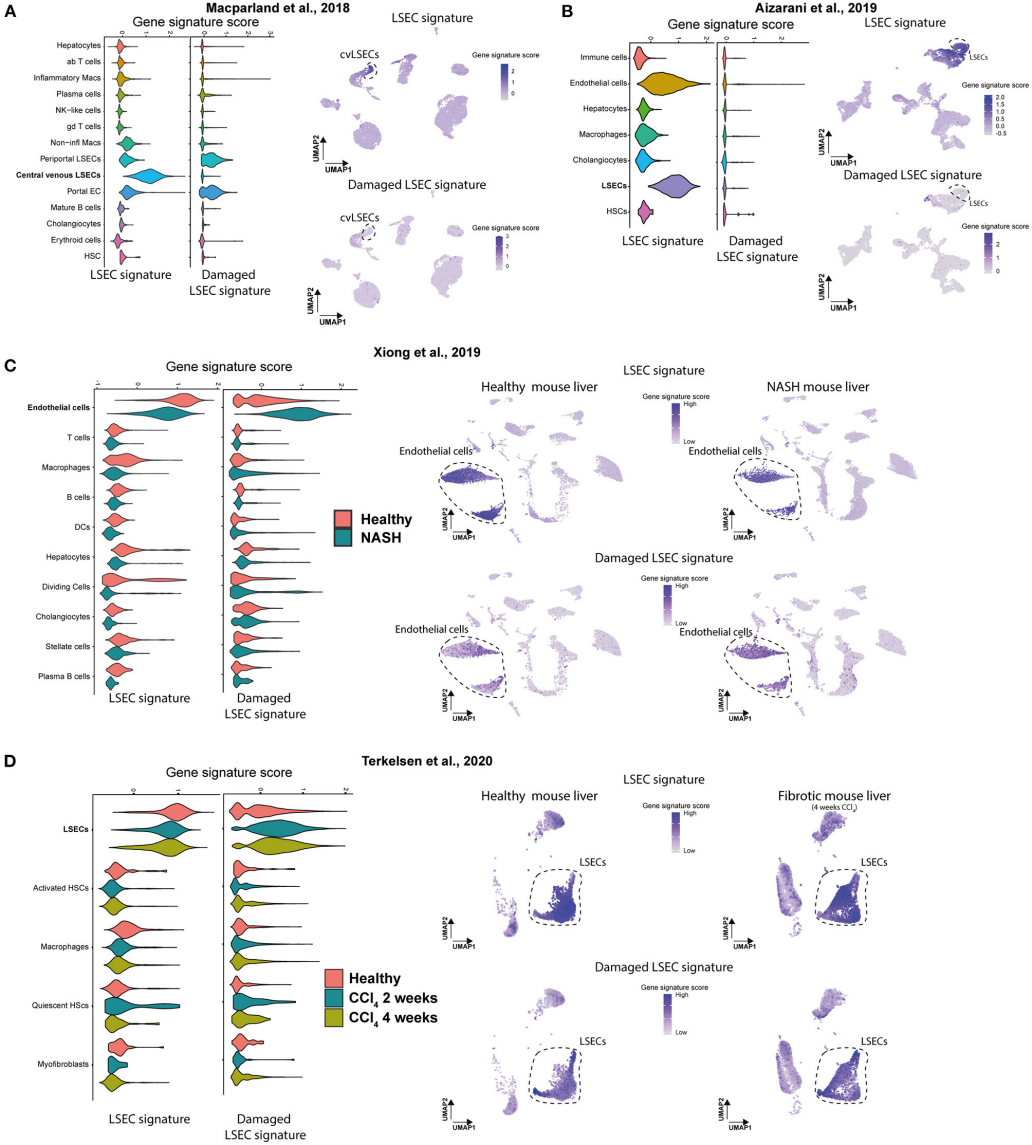
Gene signature scores of LSEC signatures in scRNAseq data of human and mouse livers. **(A,B)** Gene set enrichment score (Violin plot left) of both signatures and UMAP plot of scRNAseq data of healthy human livers with gene set enrichment score of both signatures in purple (right). Results were obtained with the use of the dataset of Macparland et al. (24) and Aizarani et al. (21). **(C,D)** Gene set enrichment score (Violin plot, left) of both signatures and UMAP plot of scRNAseq data of (NASH and fibrotic) mouse livers with gene set enrichment score of both signatures in purple (right). Results were obtained with the use of the dataset of Xiong et al. (26) and Terkelsen et al. (32).

Next, we wanted to examine if scRNAseq data sets of LSECs from healthy and diseased livers can be identified as such with these two LSEC signatures. As samples can differ quite a lot between studies due to a different definition of healthy subjects, different isolation methods, different scRNAseq approaches, different etiologies and species we validated our newly generated LSECs signatures in 4 independent scRNAseq data sets of healthy human livers (21, 24) and healthy or diseased (NASH and fibrotic) mouse livers (26, 32) (Supplementary Figure 6). Using the LSEC signature we could show a higher gene signature score in LSEC-related populations in healthy human livers compared to all other cell populations (Figures 6A,B). Moreover, the damaged LSEC signature shows a low gene signature score in all liver cell types in healthy human livers except for a slightly higher gene signature score for periportal LSECs and (portal) ECs. These results confirm that quantification of LSEC signatures (scores) can be used to identify LSECs in scRNAseq data of human healthy livers. Unfortunately, we could not validate our signatures in a different scRNAseq data set of cirrhotic patients due to the lack of publicly available human data. Next, we validated the LSEC signatures using scRNAseq data of healthy and diseased (NASH and fibrotic) mouse livers (26, 32). In both data sets, the LSEC/EC populations from control livers have a high LSEC signature score, but is also still present (but lower) in NASH and fibrotic livers. More importantly, the damaged LSEC signature score is higher in LSEC/EC population from NASH livers, and to a lesser extend in CCl_4_ livers, indicating that LSECs are damaged and can be identified in NASH and fibrotic livers using these 4 genes (Figures 6C,D). These findings demonstrate that the LSEC signatures can be used to identify and distinguish damaged LSECs from healthy LSECs in scRNAseq data of human and mouse livers.

## Discussion

LSECs are important for liver homeostasis and play a pivotal role in both acute and chronic liver injury by influencing HSCs and other cell types in the liver. ScRNAseq studies identified numerous EC populations and revealed well-established and novel LSEC markers for LSECs in healthy and disease states. However, most studies use one specific mouse model (12, 26, 32) or only human cirrhotic livers (25). In this study we sought to generate LSEC signatures that can identify healthy and damaged LSEC populations in multiple transcriptome data sets. We first focussed on genes enriched in LSEC or damaged LSEC/EC in the human liver scRNAseq data of Ramachandran et al. (25). Using these enriched human gene sets we could show that in cirrhotic livers of patients suffering from HBV, HCC, ASH, and NASH there is a clear enrichment of damaged LSECs. Subsequently, we showed that during acute liver injury in mice certain LSEC specific genes are quickly downregulated which resulted in a more specific LSEC signature that can identify healthy LSECs in mouse and human scRNAseq data. Finally, we developed a damaged LSEC signature comprised of Fabp4/5 and Vwf/a1 that can identify damaged LSECs in transcriptome data of NASH and fibrotic mouse livers.

In this study we used CCl_4_ to induce an acute liver injury and to evaluate whether the transcriptional changes that occur in LSECs in chronic liver disease already occur upon acute liver damage. After an acute liver injury we observed that LSECs quickly change their phenotype by upregulating Lyve1, by temporarily downregulating CD32b, proliferating and upregulating ECM genes after 3 days of CCl_4_. Previous studies showed that LSECs can produce a basement membrane during chronic liver injury by deposition of Collagen 4 and Laminin (52, 53, 55). Interestingly, in the data of Ramachandran et al. (25), we also see the expression of *COL4A1* and *COL4A2* mainly in the damaged LSEC/EC population (Supplementary Figure 7) indicating that primarily LSECs express *COL4A1* and *COL4A2* in chronically injured human livers. Here, we could show that Collagen 4 deposition is already initiated during acute liver injury.

Here we defined an LSEC signature that contains several well-established LSEC markers such as the scavenger receptors *STAB2, CLEC4G, CD209, MRC1*, and *CD32B* (*Fcgr2b*) but also receptors important for VEGF signaling such as *KDR* and *NRP1* (Figure 5). The expression of some of these markers (*STAB2* and *CLEC4G*) has been shown to decrease during chronic liver disease (47). Other genes in this signature are less known but have been mentioned mainly in gene profiling studies (*OIT3, NPL*) (24, 56, 57). Genes that showed a higher expression after acute liver injury in mice were not included in the LSEC signature, such as *LYVE1* and *STAB1*. However, we would like to note that these genes could still be useful markers because the induction is scaled per gene, meaning that there is an induction of expression but this induction could be insignificant if the expression of that certain gene is already very high in the LSEC population. There were several other LSEC enriched genes, such as *CLEC1B, CD14, IL33*, and *CCL6/9*, that showed an induction after acute liver injury and that have been mentioned in other gene profiling studies. This indicates that inflammation could play a role in LSECs during acute liver injury. *TIMP1* and *TIMP2*, often associated with HSCs, also show an induction. Further analysis of these genes in data from Ramachandran et al. (25) showed a strong expression of *TIMP1* and *TIMP2* in LSECs and endothelial cells from human livers indeed showing that these cells do express *TIMP1* and *TIMP2* (Supplementary Figure 8). However, TIMP1 and 2 were not expressed in LSECs or endothelial cells from scRNAseq data from Xiong et al. (26). The damaged LSEC signature contains the known capillarization marker *VWF*, and genes *VWA1, FABP4*, and *FAPB5*. Further investigation of the literature shows that protein expression of these signature genes are indeed associated with a damaged LSEC phenotype in mice and human. For example, *FAPB4*, also known as (adipocyte) fatty acid binding protein 4, was recently found to be upregulated in LSECs during liver fibrosis, can promote LSEC capillarization and is suggested to be a key regulator involved in the onset and progression of fibrosis in two liver fibrosis models in mice (58). In addition, FABP4 is also overexpressed in patients with HCC (59). Multiple studies have shown that vWF is not expressed by LSECs in healthy livers but is increased in LSECs during fibrosis in several animal models, for example after CCl_4_ treatment in mice and rats (60, 61), and NASH with or without cirrhosis in rats (62). Moreover, vWf^+^ LSECs were significantly correlated to the fibrosis stage in patients with cirrhosis (63) and a higher vWF expression has been linked to old age and pseudocapilarization (64). Targeting LSECs to alleviate fibrosis through one of these 4 genes could be an option as it was recently shown that the treatment with the *FABP4* selective inhibitor BMS309403 alleviated lipopolysaccharide induce acute liver injury and high fat diet-induced NASH in mice (65), and a knockout of *FABP4* reduces fibrosis in CCl_4_ and bile duct ligation model in mice (58).

The use of microarray or bulk-seq profiling data can mask the fact that the gene expression signal detected represents only a small portion of a total LSEC population. Few dedifferentiated or damaged LSECs could be responsible for the enrichment of the damaged LSEC/EC gene sets. To obtain more insight into the abundance of dysfunctional LSECs in human and (damaged) mouse livers, more specific LSEC gene signatures were validated in scRNAseq datasets. In this study scRNAseq datasets of different liver disease models were used; two healthy human scRNAseq data sets (21, 24) and two mouse healthy and NASH/fibrotic data sets (26, 32). The recent dataset from Su et al. (12) was not included due to a potential contamination of duplets, making incorporation of this dataset in this study problematic (data not shown). In healthy human livers, the LSEC signature separates LSECs from other liver cells, and only a low signature score is present for periportal LSECs and portal ECs when the damaged LSEC signature is used. Nevertheless, it remains difficult to separate portal and central endothelial cells from portal and central LSECs as they cluster strongly together because LSECs still express endothelial markers such as *CD31* or *CD105* even though these markers have been reported to be lower in LSECs (11, 12). In both healthy and NASH/fibrotic mouse livers, the LSEC signature was abundantly expressed even though the gene signature score is clearly lower in NASH/fibrotic livers which suggests that LSECs partly lose their phenotype in chronic liver disease. More importantly, the damaged LSEC signature had clearly a high gene signature score in all cells of the LSEC/EC population of NASH livers which indicates that all LSECs are damaged in NASH/fibrotic mouse livers. Further scRNAseq analysis of acutely injured mice or human livers would shed more light on the independent changes of different endothelial and LSEC populations and could give more insight into early mechanisms of LSEC-dysfunction or capillarization. A next step in this research could be a larger prospective sequencing effort on biopsy material of livers at different stages of chronic liver disease, or recovering from liver disease, to evaluate whether one can correlate the rise of a healthy LSEC signature to the improvement of liver fibrosis while a certain level of the damaged LSEC signature can predict progression of the liver disease. Some proteins from the damaged signature could be measured in blood and correlated to the development of fibrosis. For example FABP4 in the blood is already positively correlated to the fibrosis stage and inflammatory grade in patients with NAFLD and NASH (66). In addition, protein levels of the damaged LSEC signature genes could serve as biomarkers for the extent of LSEC damage in acute liver injury, as LSEC damage occurs in ischemia-reperfusion, drug-induced liver injury and hepatic sinusoidal obstruction syndrome (67). For instance, one study showed that FABP4 was elevated in the serum of mice with acute liver injury induced by a single injection of LPS (65). Moreover, in patients with acute liver injury and acute liver failure, vWF is elevated in the serum, but could not be correlated to poor disease outcome (68). One should note that in this study vWF levels could have been affected as blood samples were also collected after NAC administration.

To conclude, we showed that the transcriptome of LSECs transform into a cirrhotic transcriptome independent of the etiology in multiple microarray datasets from human livers. In addition, two unique LSEC signatures were developed and validated in several independent scRNAseq datasets, demonstrating that these signatures can recognize LSECs in healthy and chronically injured livers. Moreover, using several scRNAseq data sets we showed that all LSECs isolated from NASH/fibrotic mouse livers have a damaged LSEC expression profile. These results indicate that during mouse and human chronic liver disease, the change of LSECs toward a cirrhotic dysfunctional phenotype is strong and highlights the potential of LSECs as a therapeutic target for chronic liver disease.

## Data Availability Statement

The datasets presented in this study can be found in online repositories. The names of the repository/repositories and accession number(s) can be found in the article.

## Ethics Statement

The animal study was reviewed and approved by Ethical Committee of Animal Experimentation of the Vrije Universiteit Brussel.

## Author Contributions

SV and EO: conceptualization, investigation, methodology, formal analysis, validation, visualization, data curation, and writing—original draft. VD: investigation and formal analysis. NE: methodology and formal analysis. IM: investigation and methodology. LG: conceptualization, funding acquisition, data curation, and writing—review and editing. All authors contributed to the article and approved the submitted version.

## Funding

SV was supported by Fund of Scientific Research Flanders (FWO–V) junior post-doctoral fellowship (1243121N). EO was supported by Wetenschappelijk Fonds Willy Gepts of the UZ Brussel (WFWG20-23). VD was supported by FWO 1192920N. IM was supported by FWO–V senior post-doctoral fellowship 12N5419N. The work was also supported by grants awarded to LG European Union's Horizon 2020 research and innovation program under the Marie Sklodowska-Curie Grant Agreement No. 766181, project DeLIVER and FWO-V (G030616 N, G042719 N).

## Conflict of Interest

The authors declare that the research was conducted in the absence of any commercial or financial relationships that could be construed as a potential conflict of interest.

## Publisher's Note

All claims expressed in this article are solely those of the authors and do not necessarily represent those of their affiliated organizations, or those of the publisher, the editors and the reviewers. Any product that may be evaluated in this article, or claim that may be made by its manufacturer, is not guaranteed or endorsed by the publisher.
