# Supplementary material for: Gene Signatures Detect Damaged Liver Sinusoidal Endothelial Cells in Chronic Liver Diseases
Source: Front Med (Lausanne). 2021 Oct 20;8:750044. doi: 10.3389/fmed.2021.750044 (PMC8564042; doi:10.3389/fmed.2021.750044)
Supplement: Supplementary file 1 [file Data_Sheet_1.docx]

# **Supplementary data**

**
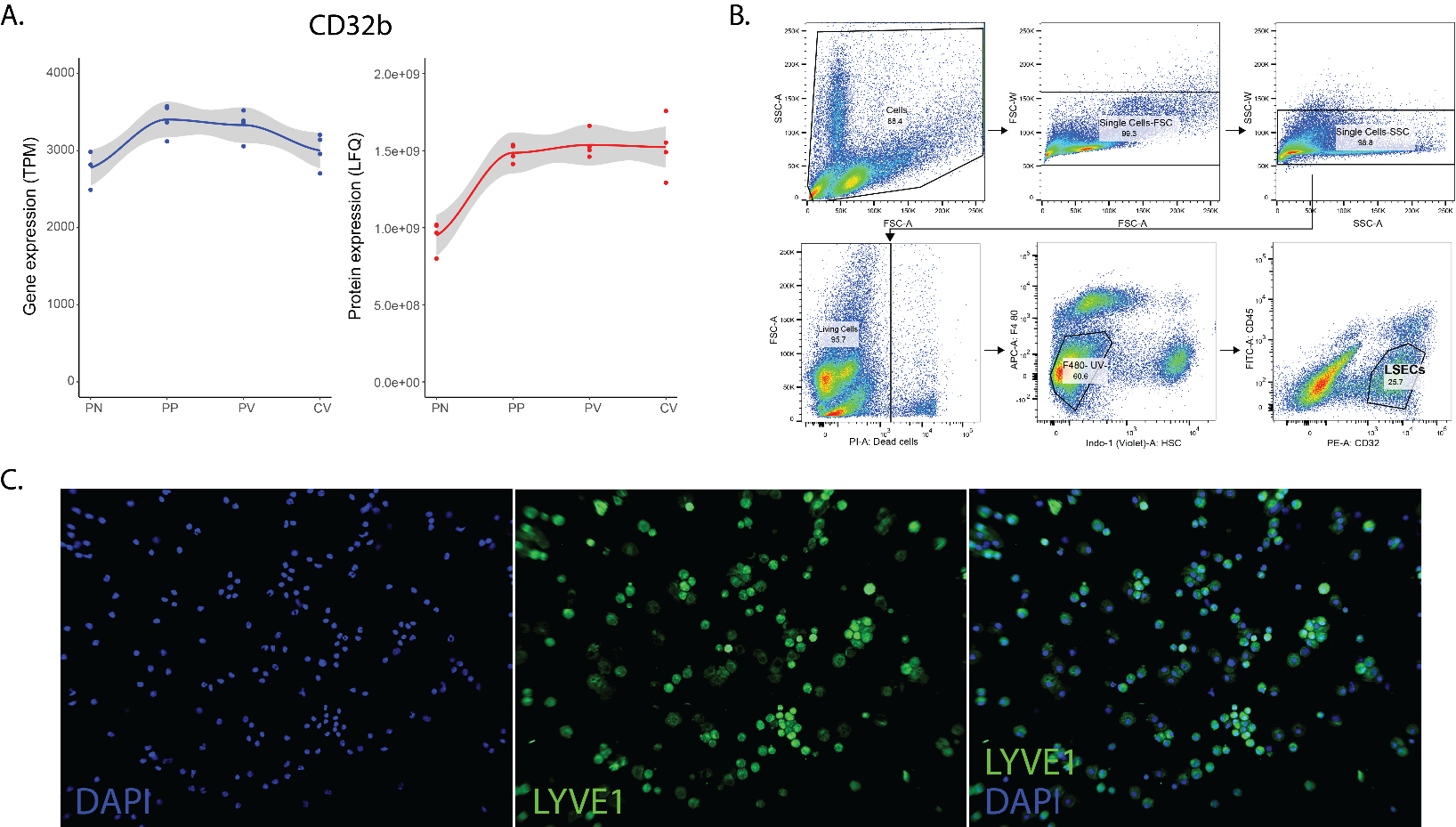
Supplementary Figure 1. Sorting strategy applied for isolation of LSECs after acute injury.** (A) mRNA (left) and protein (right) expression of CD32b in portal node (PN), peri-portal (PP), peri-central (PC), and central vein (CV) endothelial cells which shows CD32 expression in all LSECs. Data used from Inverso et al., 2021. (B) In SSC vs FCS density plot cells were selected to remove cell debris. FCS-W vs FCS-A and SSC-W vs SSC-A density plot were used to exclude doublets. Single cells positive for PI were identified as dead cells and excluded from the sort. Populations positive for CD32 and negative for F4/80 (Kupffer cells marker), UV (autofluorescence from HSCs) and CD45 (immune cells) were identified as LSECs and sorted for bulk RNA-seq. (C) Cytospins of CD32^+^UV^-^F4/80^-^CD45^-^ sorted cells were stained for Lyve1 (LSEC) and DAPI (nucleus).

**
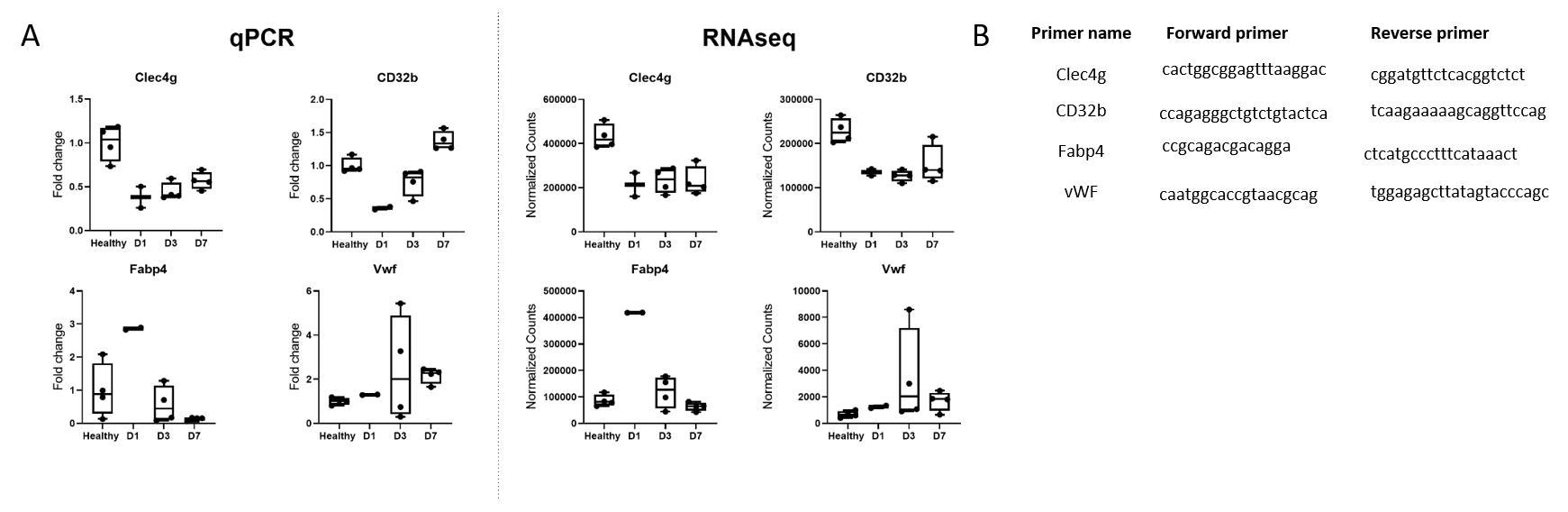
Supplementary Figure 2. Validation of RNAseq by qPCR.** (A) Gene expression of selected group of signature genes in LSECs of healthy mice and CCl_4_ treated mice. (B) Primers used for qPCR validation.


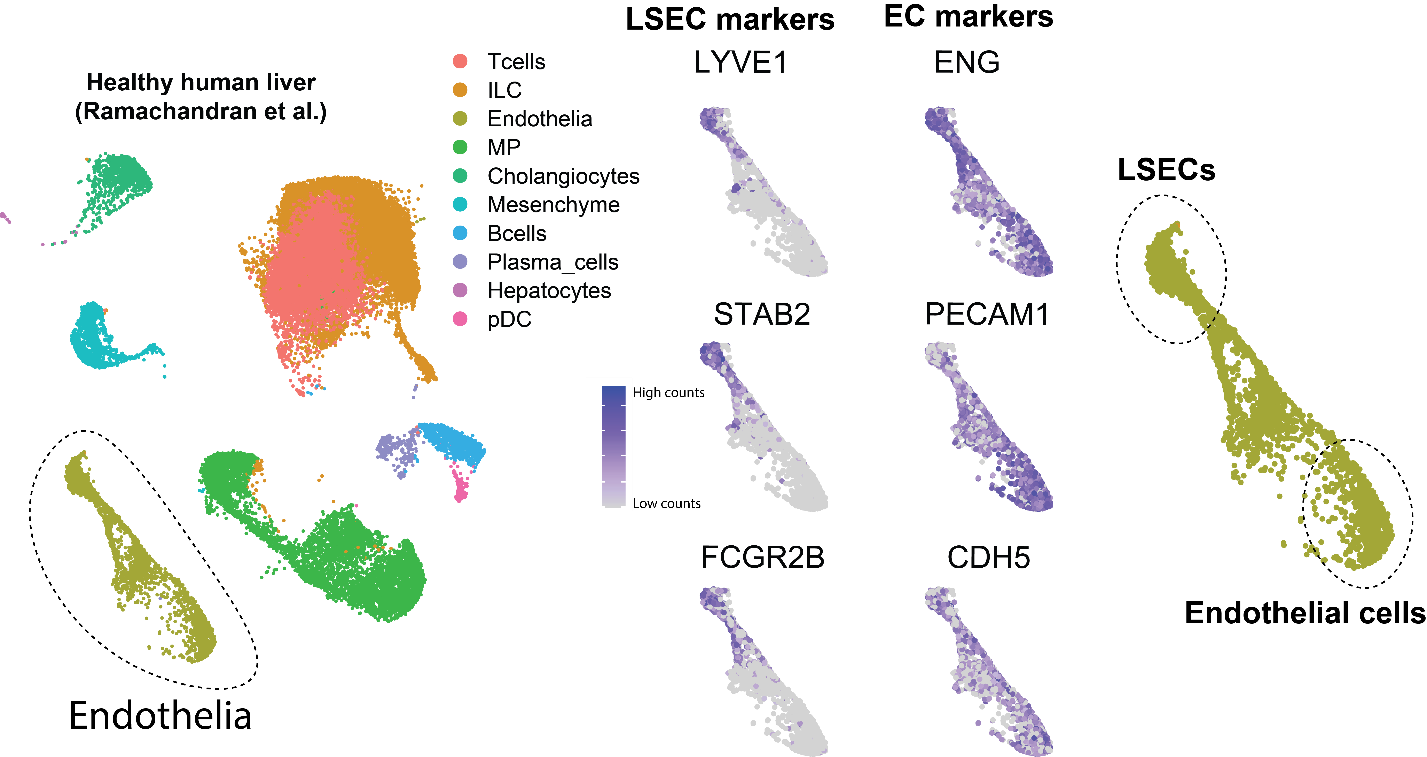


**Supplementary Figure 3. Identification of LSECs and endothelial cells in healthy human livers.** UMAP of scRNAseq data of healthy human livers with LSECs and EC marker expression depicted in purple in the endothelial cell population to identify LSECs and ECs. Data used from Ramachandran et al., 2019.


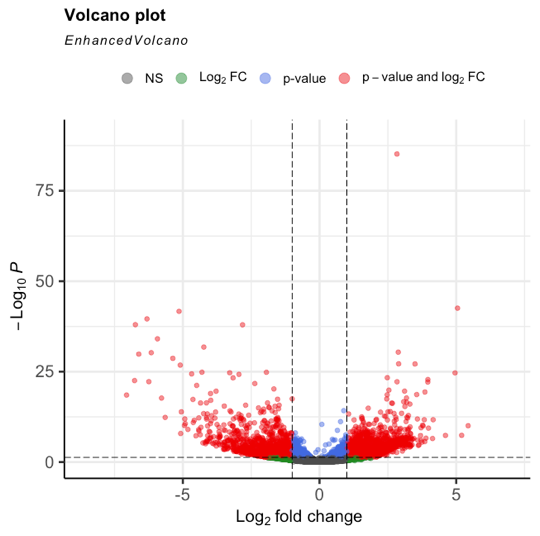


**Supplementary Figure 4. Vulcano plot of differentially expressed genes (red) in LSECs isolated from 1 day CCl_4_-treated mice vs LSECs from healthy mice.**


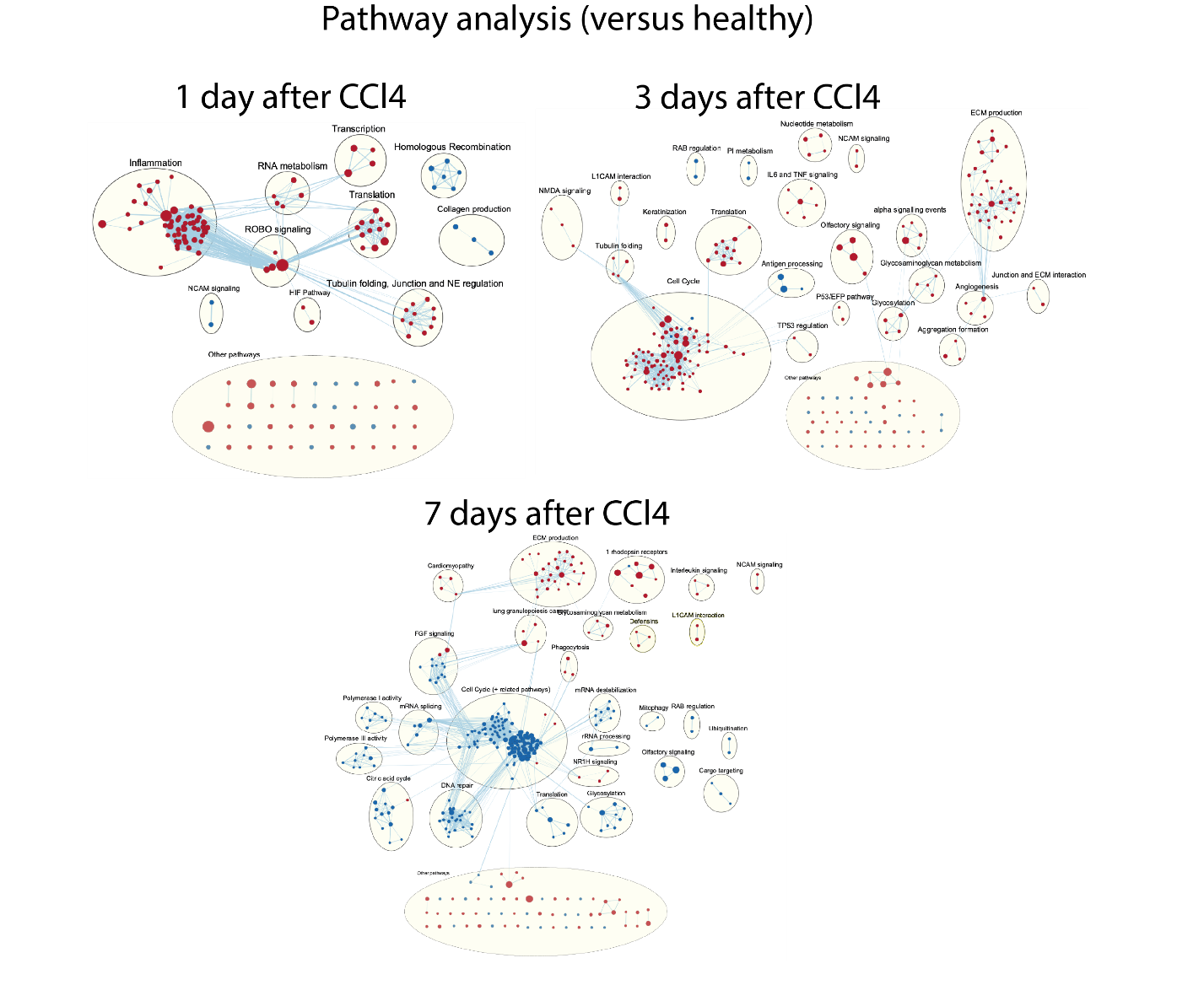


**Supplementary Figure 5. GSEA pathway analysis on LSECs from CC**l**_4_ treated livers compared to LSECs from a healthy liver.** Enrichmentmap of enriched pathways (higher enrichment=red or lower enrichment =blue) from Cytoscape in LSECs 1, 3 and 7 days after acute liver injury vs healthy liver.


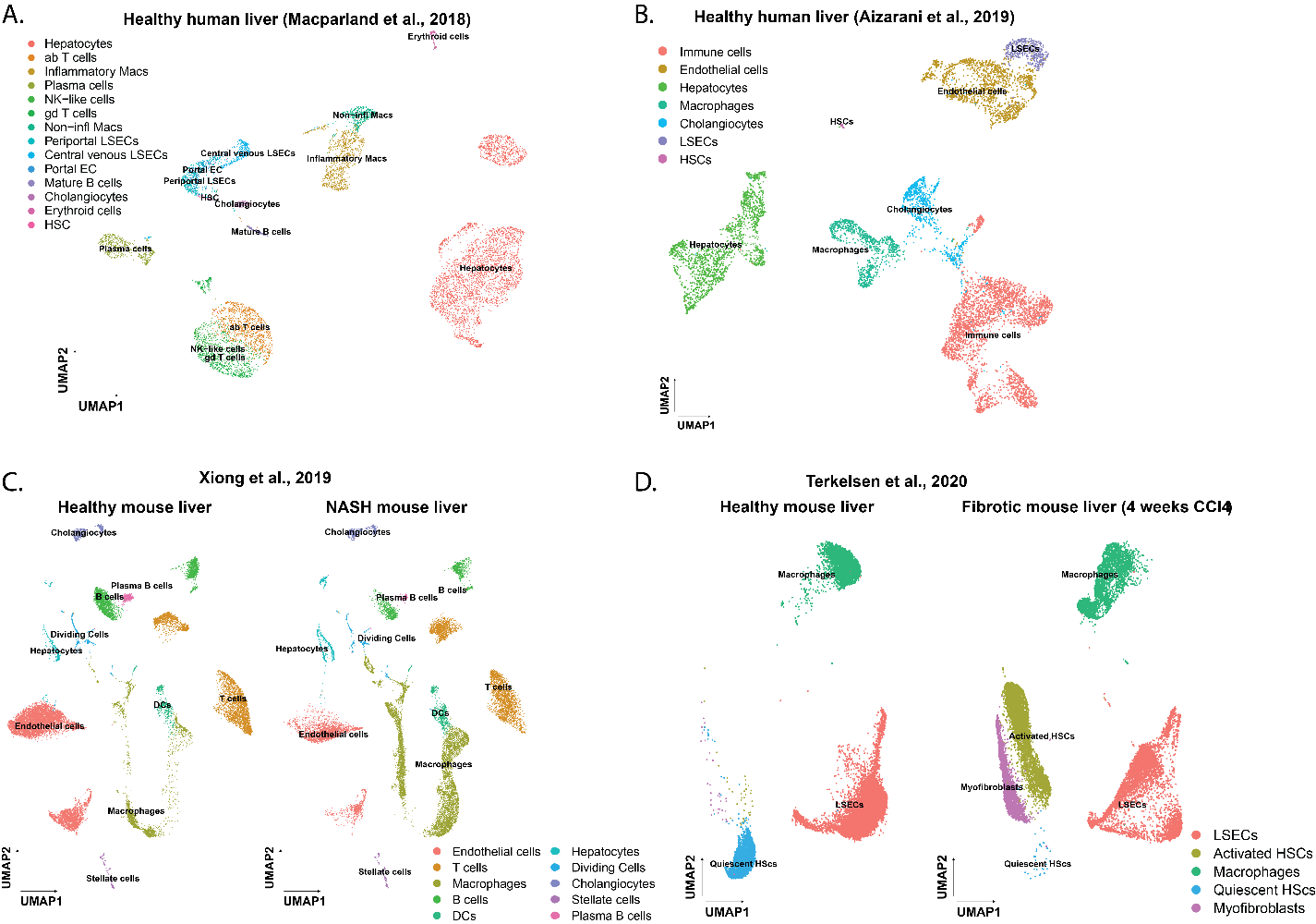


**Supplementary Figure 6. Cell populations in healthy and NASH/fibrotic mouse livers.** UMAP of scRNAseq data of healthy (A-B) and NASH/fibrotic mouse livers (C-D). Raw counts of each dataset have been normalized and clustered based on cell type specific markers used in the publication. All analysis was performed using R package Seurat.


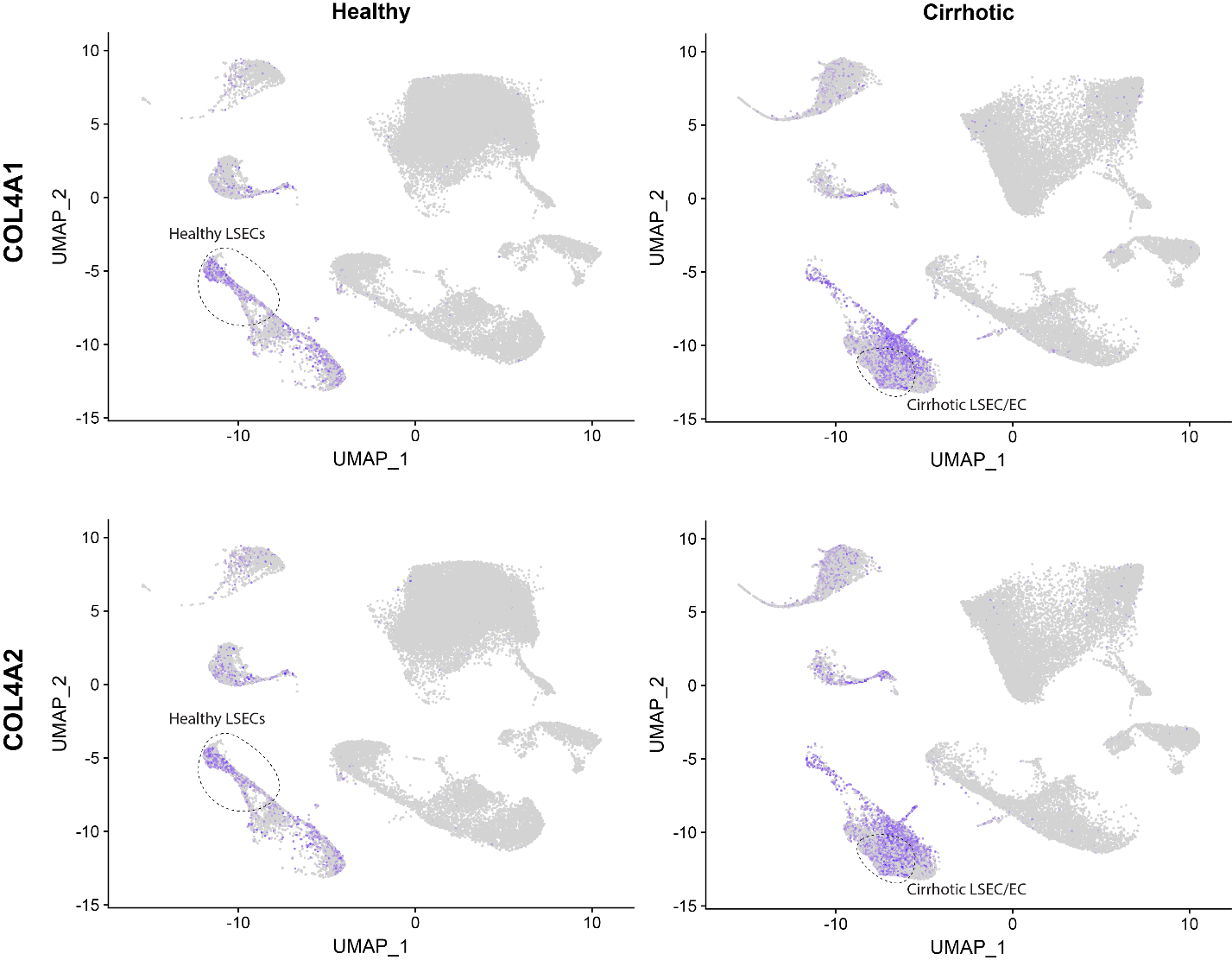
 **Supplementary Figure 7. COL4A1 and COL4A2 expression healthy and cirrhotic human livers.** UMAP of scRNAseq data of healthy and cirrhotic human livers from Ramachandran et al., 2019 with COL4A1 and COL4A2 expression depicted in purple in the LSEC and LSEC/EC population.

**
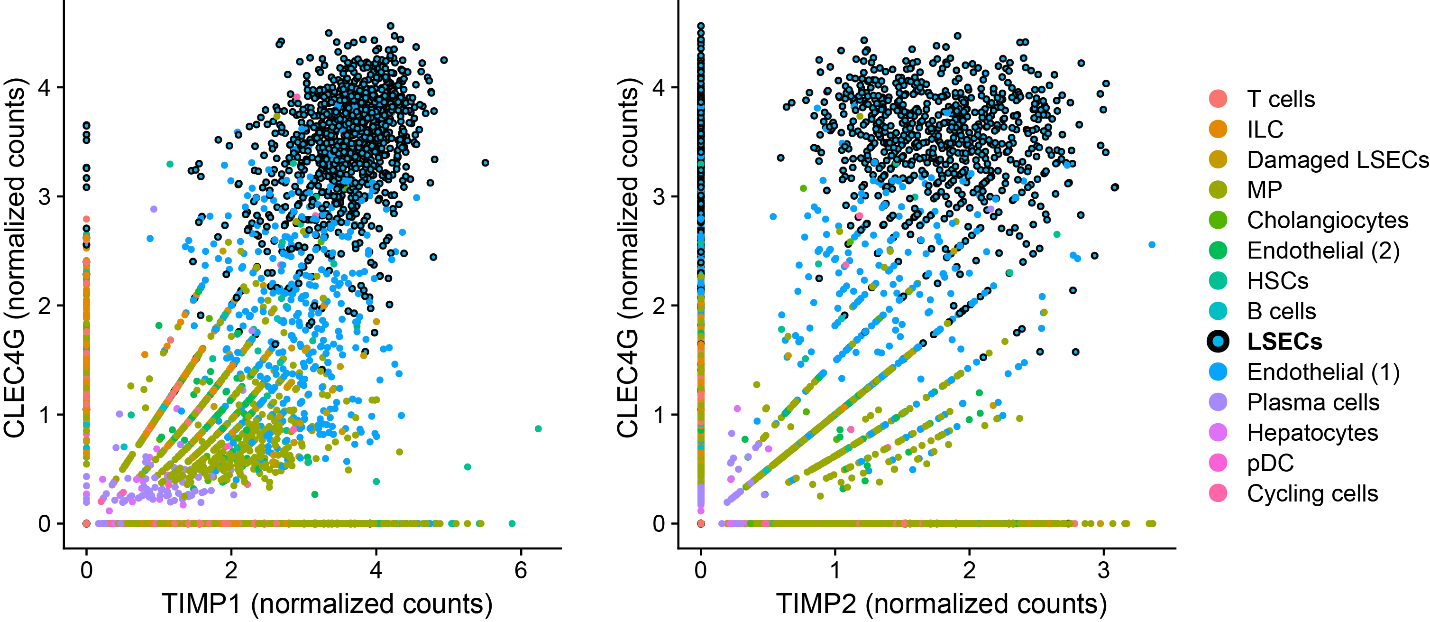
 Supplementary Figure 8. Gene expression of TIMP1/TIMP2 and CLEC4G in all liver cell types.** Normalized counts for expression of CLEC4G and TIMP1/2 in cells from healthy human liver from data Ramachandran et al., 2019. LSECs have both high expression for CLEC4G and TIMP1/2 indicating that LSECs do express TIMP1 and TIMP2.

**Supplementary Table 1.** Genes enriched in LSEC from healthy and cirrhotic human livers. Genes in bold were used for signatures.

| Genes enriched in human LSECs | | | |  |  |
| --- | --- | --- | --- | --- | --- |
| Healthy liver | | | | **Damaged liver** | |
| *Human gene symbol* | *Mouse gene symbol* | *Human gene symbol* | *Mouse gene symbol* | *Human gene symbol* | *Mouse gene symbol* |
| PLPP3 | **Plpp3** | F2R | F2r | CD34 | Cd34 |
| LIFR | **Lifr** | NID1 | Nid1 | CLEC14A | Clec14A |
| DAB2 | **Dab2** | INSR | Insr | ECSCR | Ecscr |
| IL33 | Il33 | **FCGR2B** | **Fcgr2b** | **FABP4** | **Fabp4** |
| NPL | **Npl** | FCGR2B | Fcgr3 | **FABP5** | **Fabp5** |
| STAB2 | **Stab2** | **NRP1** | **Nrp1** | FKBP1A | Fkbp1a |
| CD14 | Cd14 | CD36 | Cd36 | PECAM1 | Pecam1 |
| NTN4 | **Ntn4** | **OIT3** | **Oit3** | PLPP1 | Plpp1 |
| CD4 | Cd4 | STAB1 | Stab1 | PLVAP | Plvap |
| SEMA6A | Sema6a | ADM | Adm | RBP7 | Rbp7 |
| IL6ST | Il6st | LYVE1 | Lyve1 | RGCC | Rgcc |
| NR2F1 | Nr2f1 | TIMP2 | Timp2 | SOX18 | Sox18 |
| ECM1 | Ecm1 | TIMP1 | Timp1 | SPARCL1 | Sparcl1 |
| MAF | **Maf** | **CLEC4G** | **Clec4g** | **VWA1** | **Vwa1** |
| HSPA1A | Hspa1b | HIPK2 | Hipk2 | **VWF** | **Vwf** |
| HSPA1A | Hspa1a | RELN | Reln |  |  |
| HIBCH | **Hibch** | AKAP12 | Akap12 |  |  |
| CLEC1B | Clec1b | CRHBP | Crhbp |  |  |
| MRC1 | **Mrc1** | CTSD | Gm49369 |  |  |
| PHACTR2 | **Phactr2** | CTSD | Ctsd |  |  |
| GPR182 | **Gpr182** | TFPI2 | Tfpi2 |  |  |
| CLEC4M | **Cd209f** | PLTP | Pltp |  |  |
| CLEC4M | **Cd209g** | **SNX5** | **Snx5** |  |  |
| KDR | **Kdr** | CCL23 | Ccl9 |  |  |
| ACP5 | Acp5 | CCL23 | Ccl6 |  |  |
